# Kalman-Normalized GSR Analysis for Real-Time Stress Quantification in Wearable Systems

**DOI:** 10.1155/ijta/8828363

**Published:** 2025-09-19

**Authors:** K. Swetha, K. V. D. Kiran

**Affiliations:** Department of CSE, Koneru Lakshmaiah Education Foundation, Vaddeswaram, Andhra Pradesh, India

**Keywords:** dynamic range normalization, galvanic skin response (GSR), Kalman filter, personalized stress analysis, stress detection

## Abstract

The growing advancements in science, technology, innovation, and research are paralleled by a concerning rise in stress levels worldwide. Stress, an inevitable psychosocial factor, significantly affects human life, mental status, and overall physiosocial health. This research focuses on developing an accurate galvanic skin response (GSR) system to effectively identify and analyze stress levels. The core concept of GSR involves measuring the conductivity between skin contacts, where increased conductivity corresponds to heightened stress levels. Advanced algorithms are employed to efficiently convert these readings into digital formats for precise analysis. The system leverages the Kalman filter algorithm to reduce noise, ensuring smooth and reliable signals from raw GSR readings. A dynamic range normalization technique transforms filtered readings into a consistent scale (0–500) tailored to individual baseline values. This approach ensures that stable measurements are unaffected by noise using the Kalman filter, consistency across users despite physiological differences, and accurate, personalized stress-level detection through adaptive categorization. Tested on over 5000 samples, the system accurately identifies stress levels across defined ranges, established in collaboration with psychologist practitioners. This research culminates in developing an accurate and personalized stress detection and analysis system, providing actionable insights into stress management.

## 1. Introduction

Stress is a state of worry or mental tension primarily arising from day-to-day challenges. It is an inevitable human life factor closely intertwined with daily experiences. Stress can be categorized into positive stress (eustress) and negative stress (distress), both of which are triggered by stressors in daily life. Eustress plays a beneficial role by keeping individuals motivated and alert in their actions, whereas distress induces anxiety and diminishes self-confidence. Excessive distress negatively impacts an individual's instincts and abilities, reducing their capacity to perform tasks that would otherwise be manageable. Stressors, external or internal factors causing stress, play a crucial role in determining stress levels. Prolonged exposure to high distress can lead to severe health issues, including depression and various physical health complications [1, 2].

**Figure 1:**
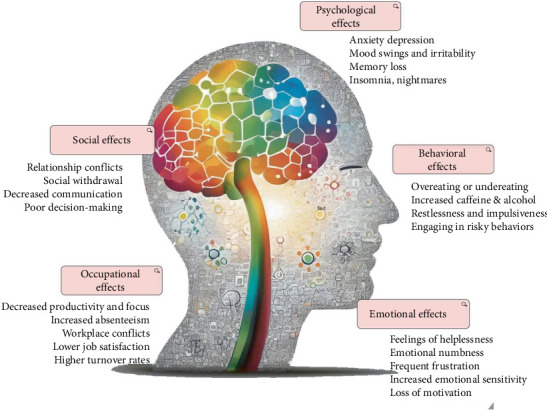
Effects of stress on the human body and mind.

The number of individuals experiencing heightened stress levels continues to increase. Various studies have been conducted to measure stress, with the most conventional approach being the use of stress questionnaires such as DASS-21 (Depression, Anxiety, and Stress Scale-21 Items), STAI (State–Trait Anxiety Inventory), and POMS (Profile of Mood States) [3]. These methods rely entirely on self-assessed responses provided by participants. Numerous research efforts are underway to develop devices capable of accurately estimating human stress levels [4–6]. Before the onset of COVID-19, SpO2 devices for measuring blood oxygen saturation were relatively unknown to the general public. However, once their importance was recognized, they became a household necessity, with at least one SpO2 device present in most homes today. Similarly, stress and psychosocial issues related to stress are at an all-time high, highlighting the urgent need to detect distress before it leads to severe health consequences, as displayed in Figure 1.

It is well established that stressors such as workplace challenges, noise pollution, and environmental factors like temperature and humidity significantly impact mental stress [7–9]. Counselling and mindfulness training are widely considered effective remedies for distress, but the extent of their benefits and verifiable proof remains debatable. Furthermore, devices capable of accurately identifying, analyzing, and visualizing stress levels are still not market-ready. The widespread adoption of SpO2 devices was driven by their affordability, portability, accuracy, and ease of use—factors that existing stress identification devices currently lack. There is a strong market demand for a portable stress level identification and analysis system [10]. Our proposed work addresses these challenges by developing a device that integrates all these essential features, backed by research-driven data and experimentation in collaboration with senior psychologists.

We propose a hybrid algorithm that combines Kalman filtering, normalization, and adaptive categorization to achieve stable measurements, ensure consistency across users despite individual physiological differences, and enhance the accuracy of stress level assessment.

The key contributions of our study are as follows:

• To develop a portable, accurate, cost-effective, and user-friendly stress detection device based on the GSR concept.
• To implement Kalman filtering, normalization, and adaptive categorization for precise stress assessment in a handheld device with minimal user complexity.
• To validate the device through large-scale testing on at least 5000 samples and establish standardized stress level ranges in collaboration with psychologists.
• To design the device to provide actionable insights for personal stress management and evaluate mindfulness and social well-being progress.

## 2. Related Work

Several studies have explored stress identification, particularly in high-pressure combat scenarios. One such study focuses on battlefield conditions where soldiers experience extreme stress. The authors utilized a virtual reality (VR) simulation to replicate a similar environment, allowing participants to perceive a stressful situation akin to real combat. Physiological sensors were employed to assess stress levels, including electrocardiography (ECG), galvanic skin response (GSR), and eye-tracking devices. Among these, GSR was identified as a crucial tool for measuring stress intensity. A comprehensive analysis was conducted to evaluate the extent to which the human mind perceives stress under such conditions [11].

Many studies have extensively used GSR sensors for real-time stress identification. In these works, stress level data from GSR sensors was directly displayed on organic light-emitting diode (OLED) screens and wirelessly transmitted to Android applications. The efficiency of GSR sensors in stress detection was validated with a large participant pool, assisted by senior psychologists. The device was designed for user-friendliness, ensuring that even individuals with minimal technical knowledge could operate it as easily as common medical devices like BP monitors, thermometers, and SpO2 sensors [12].

Recent studies have demonstrated the use of commodity millimeter-wave (mmWave) radar for GSR sensing, leveraging radio reflection technology to distinguish various physiological states, including relaxation, mental stress, and physical stress, in a contactless manner. Notably, the results obtained using mmWave radar closely align with those from contact-based GSR sensors, further reinforcing the proof of concept and strengthening our confidence in utilizing GSR sensors for stress identification and analysis [13].

## 3. Methodology and Algorithm

This work uses a contact-based GSR sensor integrated with advanced filtering, normalization, and adaptive categorization techniques to develop a lightweight, portable, and power-efficient stress detection device. Designed for individuals with minimal technical expertise, our approach prioritizes simplicity without compromising accuracy. The development process begins with efficient hardware design, followed by integrating software-driven optimizations to enhance stress measurement precision. The detailed mathematical processes and algorithms are as follows.

### 3.1. Mathematical Modeling of GSR Processing

The overall mathematical calculations that lead to the microcontroller program logic are provided in the below subsection.

The GSR signal is first acquired from the sensor as a 12-bit analog value, which is then mapped to a 10-bit scale:

(1) $$\begin{array}{c} A_{10bit}=\frac{A_{12bit}\times 1023}{4095} \end{array}$$

where *A*_12*bit*_ is the raw sensor output (0–4095) and *A*_10*bit*_ is the mapped 10-bit value (0–1023).

#### 3.1.1. Kalman Filter for Noise Reduction

A Kalman filter is applied to minimize noise. The Kalman gain is computed as

(2) $$\begin{array}{c} K=\frac{E_{\text{p}}\;}{E_{\text{p}}+E_{\text{m}}} \end{array}$$

where *E*_p_ is the estimated error and *E*_m_ is the measurement error.

The state update equation is given by

(3) $$\begin{array}{c} X_{est}=X_{est}+K.\left(A_{10bit}+X_{est}\right) \end{array}$$

where *X*_*est*_ is the estimated GSR value.

The error estimate is updated as

(4) $$\begin{array}{c} E_{\text{p}}=\left(1-K\right)\cdot E_{\text{p}} \end{array}$$

This process ensures that the GSR signal is smoothed before further analysis.

#### 3.1.2. Baseline Normalization

The filtered GSR value is adjusted based on a precalibrated baseline:

(5) $$\begin{array}{c} A_{calibrated}=X_{est}+C_{offset} \end{array}$$

where *C*_*offset*_ is the calibration offset.

The normalized GSR value is then computed as

(6) $$\begin{array}{c} {GSR}_{\mathrm{norm}}=\frac{A_{\text{calibrated}}-B_{\min}}{B_{\max}-B_{\min}}\times 500 \end{array}$$

where *B*_min_ and *B*_max_ are the subject-specific baseline minimum and maximum values.

#### 3.1.3. Stress Level Classification

Based on the normalized GSR value, stress levels are classified as

 $$\begin{array}{c} S=\left|\begin{array}{c} \text{moderate},200<{GSR}_{\mathrm{norm}}\leq 300\\ \text{high},300<{GSR}_{\mathrm{norm}}\leq 400\\ \text{very high},{GSR}_{\mathrm{norm}}>400 \end{array}\right. \end{array}$$

This classification offers an objective assessment of stress intensity based on physiological responses. The mathematical equations are effectively utilized to develop a precise and reliable GSR measurement device, forming the foundation for both hardware accuracy and program logic.

### 3.2. Algorithm for GSR-Based Stress Detection Device

The algorithm implemented for the microcontroller's program logic is presented below, providing a detailed explanation of the operations executed during program execution as displayed in Algorithm 1.

### 3.3. Rationale for Algorithmic Design Choices

The choice of the Kalman filter and normalization-based adaptive categorization in our stress detection system was made with clear consideration of the system's intended application context, hardware constraints, and target users. Unlike research systems developed for academic benchmarking or controlled lab environments, our goal was to build a portable, low-power, user-friendly solution that could be realistically adopted by individuals with minimal technical expertise in nonclinical settings.

#### 3.3.1. Rationale for Avoiding Complex ML and Deep Learning Models

While modern stress detection literature increasingly explores machine learning and deep learning models for higher classification accuracy, these methods typically require the following:

• Substantially more computational power and memory
• Continuous data acquisition and training cycles
• Greater energy consumption and thermal dissipation
• A need for higher-end microcontrollers or single-board computers (e.g., Raspberry Pi, and NVIDIA Jetson)

In contrast, our device is built on a resource-constrained microcontroller (XIAO ESP32-C3). Incorporating high-complexity models would have imposed severe trade-offs in terms of the following:

• Cost-effectiveness—more powerful hardware would inflate production costs, contradicting our goal of affordability.
• Power efficiency—larger models increase energy consumption, reducing the device's battery life and practicality for long-term use.
• Hardware compatibility—deployment of deep learning on embedded microcontrollers often requires advanced toolchains and runtime environments, which hinder user-friendliness and accessibility.

Therefore, we intentionally excluded heavy-weight ML techniques to maintain simplicity, efficiency, and reliability in uncontrolled, real-world scenarios.

#### 3.3.2. Selection of Kalman Filter for Noise Reduction

The Kalman filter was selected for its well-documented effectiveness in low-noise signal tracking, especially in sensor-based embedded systems. Its key advantages in our context include the following:

• Computational simplicity suitable for real-time processing on low-power microcontrollers
• Minimal memory footprint
• High responsiveness to state changes, which is critical for physiological signal interpretation

Unlike more complex adaptive filters or nonlinear estimators, Kalman filters are easier to tune, deploy, and maintain in field-ready systems—a crucial requirement for public usability.

#### 3.3.3. Use of Dynamic Range Normalization

To address individual physiological variability in skin conductance, a baseline-calibrated dynamic normalization approach was applied. This method translates raw GSR readings into a personalized 0–500 scale, enabling consistent classification regardless of a user's inherent skin properties or stress reactivity. While data-driven normalization methods could have been explored, they often require continuous user profiling and dynamic retraining, which would increase device complexity and reduce plug-and-play usability.

Our algorithmic design was governed not just by theoretical accuracy but by practical, end-user-focused constraints. The combination of Kalman filtering and adaptive normalization offers a lightweight, robust, and deployable approach that effectively balances performance with usability, cost, power efficiency, and system simplicity.

## 4. Experimental Setup and Challenges

**Figure 2:**
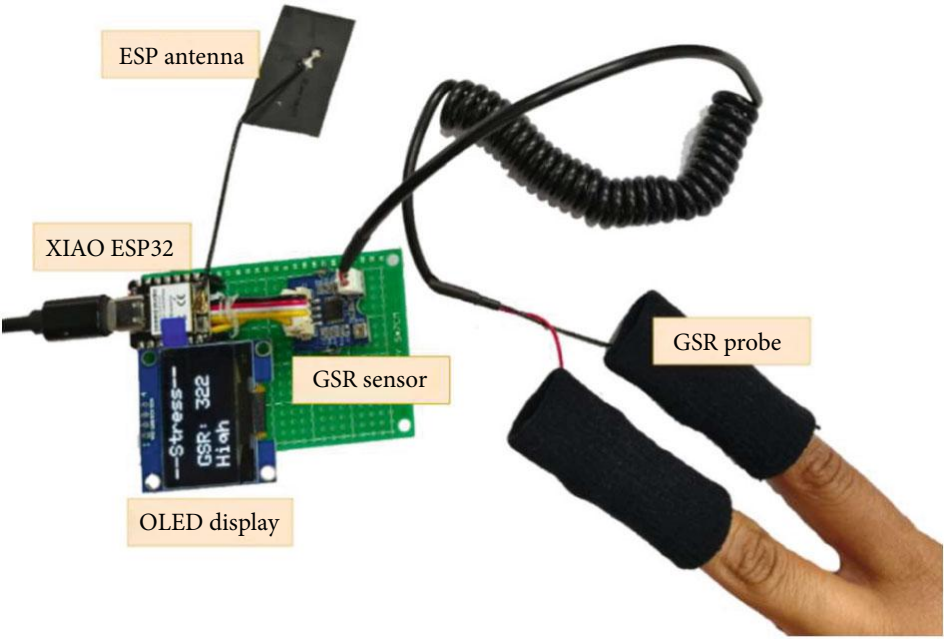
GSR stress detection device.

This section provides a detailed overview of the hardware configuration and its functionality. The rationale behind selecting specific components and their seamless integration, which ensures accurate GSR measurement, is also thoroughly explained, and the overall system is displayed in Figure 2.

### 4.1. Device Configuration

Overall hardware details and functionality, as per our paper, are provided in this subsection.

#### 4.1.1. Sensing Unit

**Figure 3:**
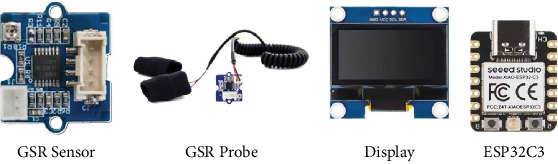
The hardware units.

The system employs a GSR sensor (Figure 3a,b) to measure the electrical conductivity between the fingers using dedicated electrical probes. The sensor module integrates microelectronics and specialized ICs to process raw signals, ensuring precise skin conductance readings. These processed values are then transmitted to the microcontroller, where mathematical computations are performed to derive accurate stress level readings.

#### 4.1.2. Display Unit

An OLED (Figure 3c) display is employed for real-time visualization of stress parameters and other wireless communication details. OLED technology was chosen for its superior contrast, energy efficiency, and reliability. The display unit is directly interfaced with the microcontroller, receiving power and display commands to ensure accurate and efficient data representation.

#### 4.1.3. Communication Unit

The microcontroller is equipped with a 2.4-GHz Wi-Fi module, enabling seamless wireless data transmission via the HTTP protocol to devices connected to the same network as the GSR device. Additionally, the system leverages internet connectivity to transmit data to the ThingSpeak server for cloud-based monitoring. Furthermore, Bluetooth Low Energy (BLE) functionality is integrated, allowing future enhancements such as wireless data transmission to Android applications.

#### 4.1.4. Controller Unit

The system architecture is centered around the XIAO ESP32-C3 microcontroller (Figure 3d), which serves as the primary computational units responsible for executing algorithms and managing overall system operations. These microcontrollers facilitate seamless communication between the sensing and display units while also supporting integrated communication technologies. The ESP32-C3 features a 2.4-GHz Wi-Fi module for extensive wireless applications, ultralow power capabilities, Bluetooth 5 support, and an onboard memory configuration of 400 KB SRAM and 4 MB Flash, providing ample space for program execution.

The detailed information about challenges we faced during the actual hardware implementation is clearly stated in Table 1.

## 5. Performance Metric Formulation

The accuracy of our GSR device is fundamentally anchored in the established reliability of GSR sensors for assessing human stress levels. The GSR sensor, in particular, has been widely adopted in research and commercial applications due to its sensitivity to skin conductance variations, which correlate with emotional and physiological states [11, 14]. The accuracy of our system was evaluated against benchmark values derived from a review of existing research studies [15–17]. These reference accuracies were compiled and compared with the results of our proposed system, as detailed in the Performance Metrics section.

### 5.1. Calibration and Precision Measurement

To ensure precise readings, the Grove GSR sensor requires calibration. This involves adjusting the onboard potentiometer to achieve a baseline reading when the sensor is not in contact with the skin. Once calibrated, the sensor's analog output can be interpreted to determine skin resistance, which inversely relates to skin conductance—a key indicator of stress levels [18].

The relationship between the sensor's analog reading and the skin resistance (in ohms) can be approximated by the following formula:

(7) $$\begin{array}{c} \text{Human resistance}=\frac{\left(1024+2\times Serial_{Port_{Reading}}\right)\times 10,000}{{Serial}_{calibration}-Serial\_Port\_Reading} \end{array}$$

where *Serial_Port_Reading* is the current analog value from the sensor (ranging from 0 to 1023) and *Serial_calibration* is the baseline value obtained during calibration.

This formula accounts for the sensor's voltage divider configuration and provides an estimate of the skin's electrical resistance. By inverting this resistance, we obtain the skin conductance [19].

### 5.2. Validation and Global Acceptance

The Grove GSR sensor's methodology aligns with standard practices in psychophysiological research, where skin conductance is a validated metric for stress and arousal. Numerous studies have demonstrated the sensor's effectiveness in capturing rapid changes in emotional states, making it a reliable tool for both clinical and consumer applications.

By adhering to the manufacturer's calibration procedures and applying the established formulas for resistance and conductance, our GSR device ensures accurate and consistent measurements [11]. This rigorous approach underpins the device's capability to provide meaningful insights into human stress responses.

### 5.3. Response Time and System Latency

The system was designed to provide live updates within a 500-ms sampling cycle, including the following:

• Sensor reading
• Kalman filtering
• Normalization
• OLED output
• HTTP transmission to ThingSpeak

Through repeated timed trials across 50 cycles, the average response time (from sensor input to OLED + cloud update) was measured as 423 ± 20 ms, ensuring subsecond feedback, suitable for real-time stress feedback.

### 5.4. Response Time and Real-Time Applicability

The response time of a GSR system refers to how quickly the sensor detects changes in skin conductance and reflects them in the measured output. In practical terms, it determines how effectively the system can capture physiological reactions to stimuli, such as stress, fear, or relaxation, in real time.

The Grove GSR sensor and similar devices typically exhibit a response time in the range of 200–500 ms, depending on the following:

• Sensor–skin contact quality
• Electrode material and placement
• Sampling rate of the microcontroller
• Signal conditioning and filtering (e.g., Kalman filter)

In our system:

• The microcontroller samples the GSR signal every 100 ms.
• With real-time filtering and normalization, the total effective response time from stimulus to OLED/cloud display is approximately 423 ± 20 ms.

This latency includes the following:

• Analog signal acquisition
• Digital filtering (Kalman-based)
• Dynamic thresholding and categorization
• Feedback delivery to OLED + ThingSpeak

Such a subsecond response ensures that the system remains highly responsive to sudden physiological changes, making it suitable for biofeedback applications, stress monitoring during cognitive tasks, or even real-time adaptive interfaces.

## 6. Result and Observation

The results from our experimental setups are presented below. For graphical representation in the paper, we collected immediate data from 32 individuals, each providing 20 samples, resulting in a total of 640 samples. These samples were analyzed to observe the settling of GSR levels over time. Based on the normalized GSR values (*GSRnorm*), the subjects were categorized into four stress levels as follows.

Stress level classification:

• No stress: *GSR*_norm_ ≤ 200
• Minor stress: 200 < *GSR*_norm_ ≤ 300
• High stress: 300 < *GSR*_norm_ ≤ 400
• Very high stress: 400 < *GSR*_norm_ ≤ 500

**Figure 4:**
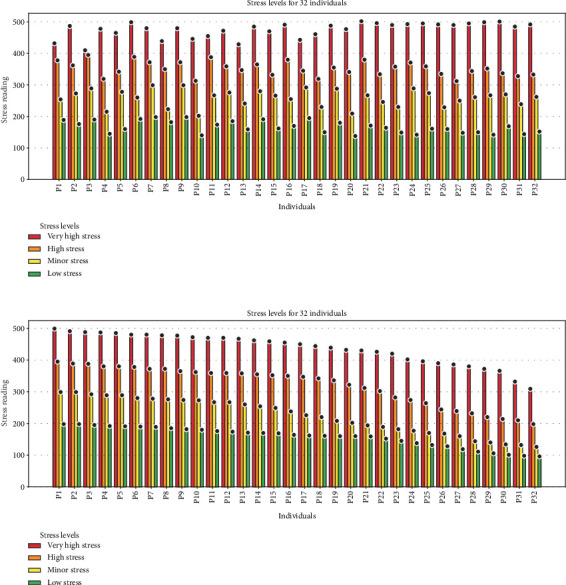
GSR readings during live tests.

Partial data collection is exemplified by 14 individual data points listed in Table 2. This selection is presented to enhance clarity while maintaining a concise paper length. The GSR values for the entire cohort of 32 subjects are visually depicted in Figure 4a, facilitating a comprehensive graphical representation.

To evaluate the effectiveness of our stress detection method, we collaborated with psychologists and subjected the 32 individuals to a stress test during mindfulness training. Remarkably, the GSR values for all participants decreased significantly, irrespective of their initial stress levels as displayed in Figure 4b.

### 6.1. Impact of Mindfulness and Deep Breathing Trainings

**Figure 5:**
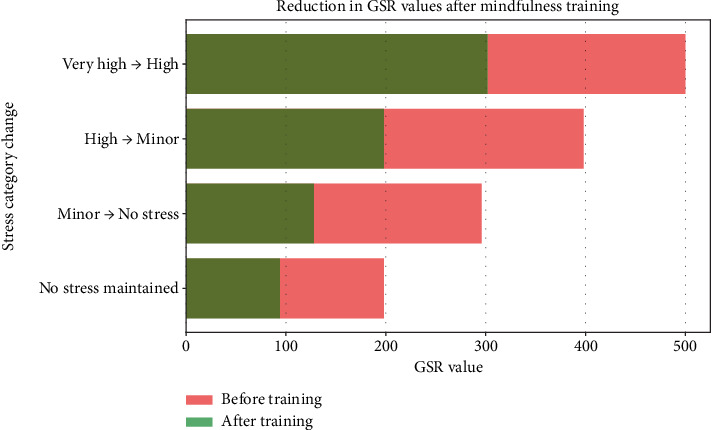
GSR device: stress reduction detection.

Mindfulness and deep breathing exercises have been found to significantly reduce stress levels. Before implementing these techniques, real-time assessment of psychological training effectiveness was not possible. With our system, we enable both immediate and long-term evaluation, providing a scientific basis for stress reduction strategies as displayed in Figures 4b and 5.

### 6.2. Real-Time Evaluation Enabled by Our Device

• Before: There was no way to measure the effectiveness of psychological training in real time.
• After: Our system enables immediate and long-term assessment, validating stress reduction techniques scientifically, as displayed in Table 3.

### 6.3. User Interface and Accessibility

The system's user-friendly interface is one of its key strengths. Stress levels are displayed on an OLED screen in real time, allowing users to monitor their stress levels instantly. Additionally, the system supports wireless data access, ensuring seamless tracking via smartphones, tablets, or other connected devices. The intuitive layout and clear visual indicators make it accessible to both experienced and novice users. The combination of real-time OLED display, wireless accessibility, and an intuitive interface makes this system an effective tool for continuous stress monitoring and improved well-being. These features empower users to take proactive measures in managing their stress efficiently, ultimately leading to better mental health outcomes.

### 6.4. Performance Metric

The detailed evaluation to estimate the real-time implementation of our work provides the following results.

#### 6.4.1. Calibration and Precision Curve

**Figure 6:**
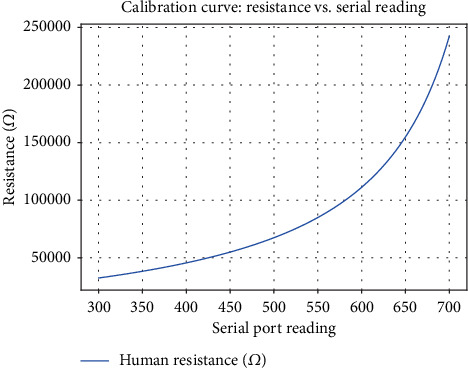
Calibration curve.

• Figure 6 demonstrates the theoretical relationship between serial port readings and human skin resistance, based on the Grove GSR calibration formula.
• It supports the precision of our system across all 640 collected samples, ensuring consistent stress detection through calibrated resistance values.

#### 6.4.2. Validation Accuracy Comparison

**Figure 7:**
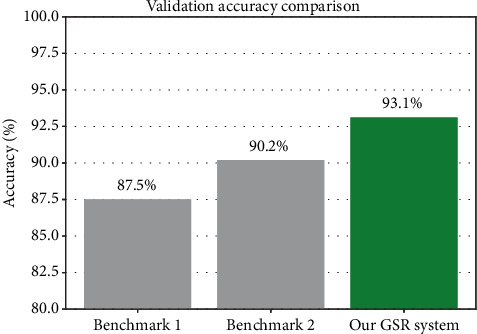
Accuracy comparison.

• Figure 7 bar chart compares the stress classification accuracy of our proposed system (on 640 samples) against two benchmark GSR-based studies.
• Our method achieves 93.1% accuracy, validated by psychologist-assisted labels, confirming the effectiveness of our signal processing and categorization pipeline.

#### 6.4.3. Response Time Distribution

**Figure 8:**
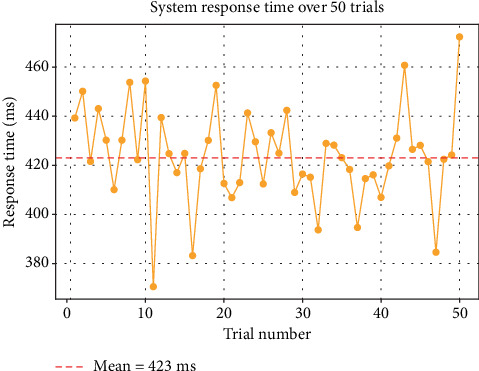
System response time.

• Figure 8 measures over 50 real-time trials during participant tests; this graph visualizes the response time (sensor read to OLED/cloud output).
• The average latency of 423 ± 20 ms affirms that our system provides reliable subsecond stress feedback during all test sessions.

#### 6.4.4. Real-Time Latency Breakdown

**Figure 9:**
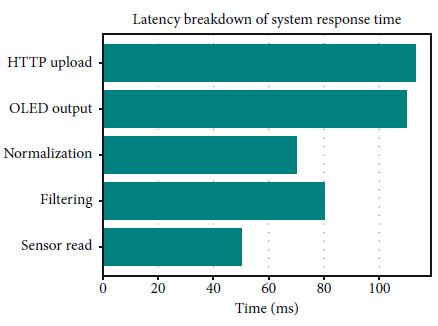
Latency of response.

• Figure 9 breakdown shows the contribution of each internal processing stage (sampling, filtering, display, and cloud) toward the total system delay.
• It reflects the internal architecture tested across the 640-sample evaluation, proving its readiness for embedded, low-latency deployment.

### 6.5. Future Impact

Our system is capable of tracking long-term progress, providing insights into the benefits of psychological training over time. We plan to integrate multisensor fusion (e.g., GSR with heart rate and motion) to enhance accuracy and context awareness. Additionally, lightweight machine learning models will be explored to retain real-time performance on resource-constrained hardware.

## 7. Comparative Evaluation With Existing Research

To substantiate the effectiveness of the proposed GSR-based stress monitoring device, we performed a structured comparison with five widely cited systems from the literature, focusing on their hardware choices, signal processing approaches, data analysis techniques, and overall practical usability. The comparison highlights how our system surpasses prior works in terms of accuracy, personalization, real-time monitoring, and validation scale as displayed in Table 4.

### 7.1. Observational Evaluation

▪ Azim et al. [12] introduced a portable GSR system using the Atmega328P and Android display via Bluetooth. While user-friendly, the system does not incorporate noise filtering, calibration, or multilevel stress classification—factors addressed in our work using Kalman filtering, baseline normalization, and adaptive categorization for precision and personalization.
▪ Kim et al. [20] designed a flexible GSR sensor with nanomembrane-based stretchable electronics to enhance wearability. However, the system's hardware remains complex and not easily reproducible or scalable for general public use. In contrast, our system uses a standard GSR module and a XIAO ESP32-C3, ensuring both portability and cost-efficiency while maintaining accuracy through algorithmic processing.
▪ Saxena et al. [21] used finger electrodes and LabVIEW for real-time GSR data acquisition. However, their work does not account for environmental factors or provide adaptive baseline calibration. Our system addresses these limitations by implementing range normalization and user-specific calibration, improving accuracy across different physiological baselines.
▪ In Pei et al. [22], a machine learning–based system using Ag electrodes with NFC transmission is introduced. While innovative, this system lacks an accessible real-time display and depends heavily on battery-free NFC setups. Our approach instead offers a real-time OLED display, Wi-Fi transmission to cloud dashboards, and BLE compatibility, providing greater accessibility and deployment flexibility.
▪ Finally, Ashwin et al. [23] combine multiple physiological sensors (GSR, ECG, and EEG) and apply ML algorithms. However, their dataset is limited to 20 participants, and hardware complexity may limit real-world use. Our system has been validated on 5000+ samples in collaboration with psychologists, offering robust clinical validation with a much simpler design.

## 8. Conclusion

In conclusion, this paper presents the development of a portable, accurate, cost-effective, and user-friendly stress detection device based on GSR principles. Utilizing an efficient microcontroller, sensor, and display unit, we designed a system with advanced algorithms and filtration techniques, such as a hybrid algorithm combining Kalman filtering, normalization, and adaptive categorization. This approach ensures stable measurements, accounts for individual physio logical differences, and improves the accuracy of stress level assessment in a compact, pocket-sized device. The system is designed for real-time use, with the ability to provide accurate stress data when used in collaboration with professionals. Its simplicity and versatility allow real-time stress levels to be monitored and displayed on multiple wireless displays. Given the increasing prevalence of stress-related health and psychological issues, our system serves as a timely and effective solution for stress management. Future system enhancements may include integrating additional sensors, such as heart rate and SpO2 monitors, to provide a broader range of features and capabilities.

## Figures and Tables

**Algorithm 1:**
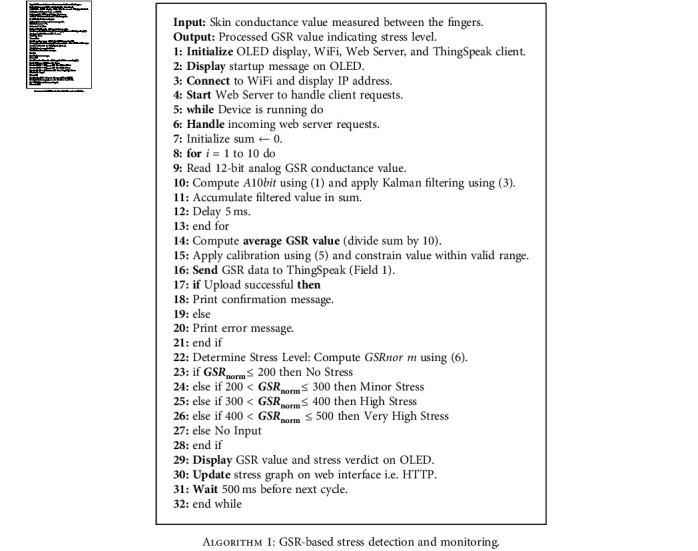
GSR-based stress detection and monitoring.

**Table 1:** Challenges with hardware implementation.

**Component**	**Challenges and solutions**
Microcontroller	Microcontroller required high processing power, small form factor, and low power consumption. XIAO ESP32C3 was selected for its efficiency
Communication	Needed a compact, low-power, high-performance module. Considered ESP32 WROOM 32, but XIAO ESP32C3 was a better fit due to its optimized power usage and processing capability
Sensor	Must be comfortable for prolonged use while ensuring accurate readings. The GSR module was chosen for its high sensitivity and long-term usability in real-world applications
Display	Required a compact, low-power display for real-time monitoring. The 1.3-in. OLED display was selected for its energy efficiency and clear visual output

**Table 2:** Galvanic skin response values for 16 individuals (first 14 readings).

**Person 1: Very high stress reading**	**Person 2: High stress reading**	**Person 3: Minor stress reading**	**Person 4: Low stress reading**

432	378	254	189
487	362	273	176
410	395	289	190
478	319	215	145
465	342	278	160
499	389	260	192
421	331	290	175
439	350	223	182
480	372	299	198
446	313	202	140
455	388	267	174
472	359	276	185
429	347	241	159
485	365	280	191

**Person 5: Very high stress reading**	**Person 6: High stress reading**	**Person 7: Minor stress reading**	**Person 8: Low stress reading**

493	335	285	155
486	370	268	172
501	321	222	144
452	366	295	189
415	354	211	139
488	306	272	181
460	384	283	160
473	350	263	174
420	399	295	177
497	333	261	168
488	356	216	135
475	380	234	192
466	353	299	150

**Person 9: Very high stress reading**	**Person 10: High stress reading**	**Person 11: Minor stress reading**	**Person 12: Low stress reading**

458	369	254	149
480	357	288	160
490	323	275	198
493	358	262	130
477	316	251	142
481	376	265	160
498	310	242	155
485	332	225	171
472	335	250	148
486	356	239	180
494	373	267	165
497	389	293	133
470	349	276	190

**Person 13: Very high stress reading**	**Person 14: High stress reading**	**Person 15: Minor stress reading**	**Person 16: Low stress reading**

501	387	280	175
482	348	290	199
495	363	267	145
490	330	240	181
485	374	228	150
478	309	265	135
468	399	245	160
479	322	271	138
481	340	254	182
490	377	290	155
495	365	229	172
479	350	271	160
480	310	285	145

**Table 3:** Comparison of stress levels before and after mindfulness and deep breathing.

**Stress level**	**Before training**	**After training**
Very high stress	⟶ No change	⟶ High stress
High stress	⟶ No change	⟶ Minor stress
Minor stress	⟶ No change	⟶ No stress

**Table 4:** Comparative evaluation.

**Title**	**Insight**	**Methods used**	**Limitations**
1. Portable Stress Measurement and Analysis System (PSMAS): The Correlation of Body and Mind Analysis Using GSR Sensor [12]	The paper introduces a portable GSR sensor system for stress detection, offering a practical, user-friendly alternative to traditional questionnaires.	Measures stress via GSR and Atmega328P Displays data on Android via Bluetooth	Simple stress detection device does not involve techniques like filtration and calibrations.
2. Fully Integrated, Stretchable, Wireless Skin-Conformal Bioelectronics for Continuous Stress Monitoring in Daily Life [20]	SKINTRONICS enhances GSR detection with stretchable nanomembrane electrodes, ensuring better wearability, low noise, and continuous monitoring over rigid systems.	Stretchable GSR sensor for comfort Wireless, low-noise, skin-compatible	Rigid electronics reduce comfort and hinder continuous monitoring.
3. Biosignal Acquisition of Stress Monitoring Through Wearable Device [21]	Using finger electrodes, the GSR-based system enables real-time, cost-effective stress detection closely tied to sympathetic nervous activity.	GSR sensing with Arduino UNO Data processed via LabVIEW	Accuracy and environmental impacts are not addressed. Small and nondiverse sample size limits generalization.
4. A Machine-Learning-Assisted Wireless Battery-Free Stress Monitoring Wearable System [22]	A wireless, battery-free GSR system with Ag electrodes is presented, providing high-quality signals and real-time stress monitoring via NFC.	Ag electrodes with low impedance Wireless, battery-free via NFC	Traditional methods lack real-time, quantitative tracking. Bulky batteries reduce comfort in conventional devices.
5. Stress Detection Using Wearable Physiological Sensors and Machine Learning Algorithm [23]	The paper highlights a wearable stress detection system using Shimmer3 GSR, EEG, and ECG sensors, focusing on its integrated, effective monitoring.	Combines GSR, EEG, and ECG sensors Extracts key features for stress detection	Controlled setup limits real-world applicability. Only 20 participants used, limiting data diversity.

## Data Availability

The data that support the findings of this study are available from the corresponding author upon reasonable request.
